# Carbonized Hemp Fiber for Use in Composites

**DOI:** 10.3390/ma18112509

**Published:** 2025-05-27

**Authors:** Sodiq B. Yusuf, Michael R. Maughan, Armando G. McDonald

**Affiliations:** 1 Forest and Sustainable Products Program, Department of Forest, Rangeland and Fire Sciences, University of Idaho, Moscow, ID 83844, USA; 2 Department of Mechanical Engineering, University of Idaho, Moscow, ID 83844, USA; maughan@uidaho.edu

**Keywords:** hemp fiber, carbonization, phenol resorcinol formaldehyde, wood, composites

## Abstract

This study investigates the use of carbonized hemp fiber (CHF) as a reinforcement for phenol resorcinol formaldehyde (PRF)-based fiber composites. The hemp fiber was carbonized slowly up to 1000 °C under N_2_ with a yield of 18%. Compression-molded composites were prepared with CHF and then compared to hemp (HF) and wood fiber (WF) at 0 to 50% loading with PRF resin. The flow characteristics of the uncured composites were determined by dynamic rheology and showed pseudoplastic behavior; the composites show promise as extrudable materials. The flexural strength of the HF composites (69 MPa for 40% HF) was higher than the CHF composites. The thermal stability of the composites was determined by thermogravimetric analysis (TGA), and the CHF composites were more stable than the HF and WF composites. Carbonization was shown to enhance both the thermal stability and the hydrophobicity of the composites, which is expected to lead to less susceptibility to weathering and biological attack. Formulations of 50% WF, 50% CHF, and 30% HF fiber loadings with PRF were able to be extruded into rods. Extruded CHF composites showed better mechanical properties than the HF and WF composites.

## 1. Introduction

The need for biobased and sustainable sources of carbon fiber (CF) to replace petroleum-based CF has shifted research attention to long natural fibers [1]. The United States Department of Energy have proposed that the use of CF will significantly decrease domestic vehicle fuel consumption by decreasing car weight [2]; however, for the automotive industry to fully benefit from CF, the material must be obtained from renewable sources [3]. Thus, there is an opportunity for long carbonized natural fibers, derived from hemp, to be produced and then used in thermoset composite materials.

Fast-growing short-rotation crops, such as hemp, yield more biomass than wood on a per-area basis [4]. Hemp can have up to a 14% higher yield than birch wood and takes 100 days to grow to a stage where it can produce fibers, while wood takes many years to reach such a stage [5,6]. Fiber from hemp can also be used as reinforcement in polymer matrix composites and as substitute for glass or carbon fiber, due to its strength and stiffness [7,8]. Bambach reported that the mechanical properties of hemp fibers (HF) were comparable to those of synthetic glass fibers [8].

Hemp fiber (HF) was reported to be the most widely researched reinforcement for polymer composites in more than a millennium [9,10]. This is due to its excellent mechanical properties, eco-friendliness, low density, and high specific strength and stiffness [9]. In 1941, HF was used in automobiles and was reported to withstand impacts 10 times stronger than comparable metal panels [11]. Bambach compared the properties of synthetic fiber, HF, and carbon fiber composites and concluded that HF composites were comparable to glass fiber composites in terms of compression strength and stiffness [8]. HF also improved the tensile strength, flexural strength, and notched and unnotched impact strength of polypropylene by 22%, 8%, 24%, and 82%, respectively [12]. Misnon compared HF and wood reinforcement and concluded that HF-reinforced vinyl ester can be used as alternative to wood and engineered wood products, based on their mechanical properties [13]. The life-cycle assessment of HF-reinforced epoxy-based composites for potential applications in aircraft and electric scooters shows less of an environmental footprint when compared to fossil-based fibers [14]. HF has been researched extensively, using various resins.

Phenol resorcinol formaldehyde (PRF), a cold-setting thermoset resin, is used widely in structural applications, mainly due to its strong mechanical performance, durability, and weather- and moisture-resistant qualities [15,16,17,18,19]. PRF has many applications, such as in glulam beams and in the automotive, electronics, and aerospace industries [20]. In previous research, PRF-wood fiber (WF) composites were extruded and cured under ambient conditions, showing their potential for use for additive manufacturing (AM) applications [18]. AM has shown great potential for producing composites that are reinforced with natural fibers, mainly due to its design freedom, lower labor demands, and material flexibility [21]. Three-dimensional (3D) printed natural fiber composites (NFC) demonstrate better mechanical properties than compression-molded composites because the fiber and the matrix mix together better during extrusion [22]. For example, the mixability of the fibers and matrix is important to maintain strength and prevent failure due to non-uniformity or porosity [23]. The main technology used for the AM of NFC is fused deposition modeling. A major drawback to the AM of thermoplastic glass-fiber composites was difficulty in extruding the material at high fiber-loading percentages (<30%) due to poor printability [24,25]. Fine hemp hurd particles were successfully printed at loadings of 40% and were shown to improve the mechanical properties of biobased thermoplastics, polylactic acid (PLA), and polybutylene adipate co-terephthalate (PBAT) [26]. Unfortunately, PLA has a glass transition temperature (T_g_) of about 70 °C, and in hot weather, PLA-composites can deform. Therefore, there is a need for high-fiber-loaded thermoset AM composites, which have high T_g_ values and do not deform under hot conditions; meeting this need forms the basis of the current study.

This study’s goal was to prepare long carbonized hemp fibers (CHF) and evaluate them for use in composite materials, in comparison with HF and WF. Fiber composites (0–50% fiber content) were initially produced by compression molding, using a PRF resin. The rheological, curing, water-soak, viscoelastic, and flexural properties were determined and discussed. Extrusion trials of WF, CHF, and HF–PRF resin composites were also performed to assess their suitability for AM, since these materials can be deposited by extrusion, and their mechanical properties compared with that of compression-molded specimens.

## 2. Materials and Methods

### 2.1. Materials

Hemp rope (6 mm diameter, Bean Products Inc., Chicago, IL, USA) was cut into 3–5 mm fiber lengths using a paper guillotine to make HF. To make CHF, hemp rope (6 mm dia, 50–70 g, in 8 batches) was placed in a stainless-steel boat and inserted into a quartz (75 mm dia × 1000 mm) tube furnace (Lindberg model 55322-3 with an Omega CN7200 ramp/soak temperature controller, Bridgeport, NJ, USA). The rope was then thermally processed as follows: (i) stabilized at 21 °C for 30 min; (ii) heated to 250 °C at 0.5 °C/min and kept for 60 min in air; (iii) heated from 250 °C to 1000 °C at 1 °C/min under nitrogen (300 mL/min, Dakota Instruments mass flow controller, Orangeburg, NY, USA); then (iv) cooled to ambient temperature under nitrogen and the CHF gravimetric yield determined. Wood-mill residues were obtained from Plummer Forests Products (Post Falls, ID, USA) and classified by passing through a standard 40-mesh screen, with the fibers labeled as WF. PRF resin (Cascophen 4001-8 resin and Cascoset 5830 E hardener at a 2.5:1 ratio) was obtained from Hexion (Columbus, OH, USA).

### 2.2. Composite Fabrication by Compression Molding

WF, HF, and CHF, at loadings of 10 to 50%, were blended with PRF resin and hardener on a dry-weight basis in a coffee grinder for 2 min. The mixture was then transferred to a 75 mm pellet die set and pressed into a disc at 160 °C for 7 min under a 1-tonne load (PHI hydraulic press, City of Industry, CA, USA), based on previous research [18]. After pressing, the composites were kept flat at room temperature for 24 h before being transferred to an oven at 105 °C for 24 h to post-cure [18].

### 2.3. Composites Fabrication by Extrusion

The fiber–resin blends were evaluated for their extrudability using a single screw extruder ((RobotDigg SJ20, Shanghai, China), 250 W motor, 17 rpm, 20 mm Ø barrel, 200 mm screw length, 8.5 mm pitch, and a 9 mm Ø die). The WF, HF, and CHF samples at loadings of 30 to 50% were then blended with PRF resin and hardener on a dry-weight basis (50 g batch size) in a coffee grinder for 2 min. The mix was introduced into the extruder barrel and extrusion was carried out for 10 min. The extruder barrel was cooled with ice water, pumped through a tightly wound copper tubing coil to prevent the resin pre-curing. For successful extrusion, the extruded rods were then cold-pressed (PHI hydraulic press, City of Industry, CA, USA) between 2 aluminum caul plates (300 × 300 × 2 mm^3^) with 3.1 mm stops to obtain flat ribbons. The flattened ribbons were then cured in an oven at 105 °C for 24 h, based on a previous study [18].

### 2.4. Fiber and Composite Characterization

The density of the fibers weas determined using an ultra-pycnometer 1000 (Quantachrome, Boynton Beach, FL, USA) in nitrogen at room temperature. The specific surface area analysis of the fibers was determined using the Brunauer–Emmett–Teller (BET) method on 0.5 g of the sample, in duplicate, using a FlowSorb 2300 instrument (Micromeritics, Norcross, GA, USA) according to ASTM D6556 [27]. Elemental (C and N) analysis was performed at the University of Idaho Analytical Laboratory. Particle size analysis of the CHF and WF was performed on a Bettersizer-2600 instrument (Costa Mesa, CA, USA). The fiber length for HF was determined manually using a dissecting microscope on 50 fibers. FTIR spectroscopy was performed on a Nicolet-iS5 spectrometer (Thermo-Scientific, Madison, WI, USA) with an attenuated total reflectance (ATR) accessory, using a ZnSe crystal for WF and HF and a Ge crystal for CHF. The spectra were ATR- and baseline-corrected using the Omnic v9.8.3 software. Lignin syringyl-to-guaiacyl ratios (S/G) were determined from the relative band heights of 1460 cm^−1^/1512 cm^−1^. Raman spectra of the CHF samples were recorded on an Alpha 300R Raman microscope (Witec, Ulm, Germany) at 532 nm of excitation, with 0.5 s acquisition time, 10 scans per replicate, and 5 replicates. The spectra were averaged and baseline-corrected, and the 1360 cm^−1^ and 1600 cm^−1^ bands were fitted using IGOR Pro v8.04 (Wavemetrics, Portland, OR, USA). The ratio of D (disordered, 1360 cm^−1^)/G (graphitic, 1600 cm^−1^) band intensities (I_D_/I_G_) was used to calculate the content of disordered carbon in the CHF. Scanning electron microscopy (SEM) was performed on the fibers after a gold coating was applied, using a Zeiss Leo Gemini (Dublin, CA, USA) field emission SEM at 5–8 kV.

The rheological properties (complex viscosity (η*), storage modulus (G’), and Tan (δ) were tested on freshly blended composite discs (2 mm × 25 mm Ø) and neat resins, using a Discovery hybrid rheometer (DHR2, TA instruments, New Castle, DE, USA) between two serrated parallel plates (for fiber–resin blends) and disposable aluminum plates (for the PRF resin). To prepare composite samples (2 g batches), an herb grinder was used for mixing (2 min) and for preparing the dynamic rheology specimens (2.0 mm (h) × 25 mm Ø) by cold-pressing them in a 25 mm Ø pellet die with a 0.45-tonne load. Measurements were determined in an isothermal (30 °C) experiment with 0.1% strain and a frequency sweep from 0.01 to 100 Hz.

The thermal stability of wet and cured samples (5 mg) was determined using a Perkin-Elmer TGA-7 instrument (Shelton, CT, USA) under nitrogen (30 mL/min) from 30 °C to 900 °C at 20 °C/min, while the data were analyzed using the Pyris v13.3.1 software. The viscoelastic properties of the cured WF-, HF-, and CHF-based composites (3 mm × 4 mm × 15 mm) were investigated at 3 °C/min using the 3-point bend mode (15 mm span, 1 Hz, and 0.1% strain) in duplicate using a Perkin-Elmer DMA-7 instrument (Shelton, CT, USA). Compression mode (5 mm disc, 1 Hz, and 0.1% strain) was employed for cured PRF in duplicate at a rate of 3 °C/min from 30 °C to 350 °C. Pyris v13.3 was used to evaluate the DMA data.

The flexural properties of the cured samples, which were cut into 5 rectangular specimens (63.5 mm × 13.5 mm × 3 mm), were determined according to ASTM D790 [28] using a Mecmesin MultiTest 2.5-dV (PPT Group, Slinfold, UK) testing machine (2.5 kN load cell, 48 mm span, and a cross-head speed of 1.1 mm/min). Data were collected and analyzed using Mecmesin VecterPro V6.1.0.0 software (PPT Group, Slinfold, UK).

The thickness swell (TS) and water absorption (WA) of the cured dry specimens (20 mm × 20 mm × 3 mm) were determined at 24 h and 72 h from submerged samples in distilled water at room temperature. The wet test blocks were removed and blotted with paper towels to remove excess water prior to measuring their dimensions and weight. The percentage weight gain and swelling thickness of the composites were calculated from the initial and final weights and dimensions, respectively.

## 3. Results

### 3.1. Fiber Characterization

**Figure 1:**
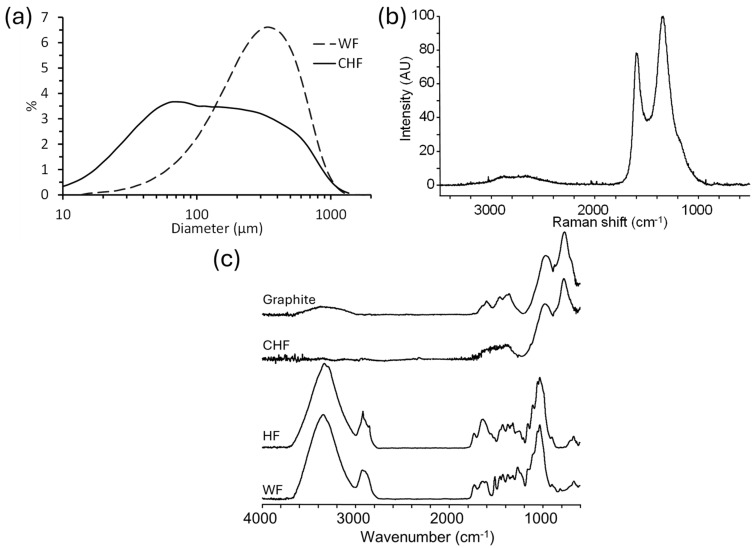
(**a**) Particle size analysis of CHF and WF, (**b**) Raman spectrum of CHF, and (**c**) the FTIR spectra of WF, HF, CHF, and graphite.

The density of HF and WF were 1.50 and 1.43 g/cm^3^, respectively. These density values are within the range given in the literature for wood and hemp [29]. The carbonization of HF, using similar procedures to those used in producing carbon fiber, resulted in a yield of 18 ± 2% CHF. The yield was within the range (10–30%) for the carbonization of cellulose to CF [30] but was lower than when using lignin carbonization (42%) [31]. The density of CHF was 1.09 g/cm^3^. The C content of HF, WF, and CHF were, respectively, 44%, 50%, and 88%, which clearly shows, as expected, that the C content doubled upon the carbonization of HF. The WF and CHF average particle sizes were 284 and 118 µm, respectively (Figure 1a). The average fiber length for HF was 3400 µm. The surface areas of the WF, HF and CHF samples, respectively, were 1.01, 0.75, and 203 m^2^/g. These results showed that the carbonization of HF increased its surface area by two orders of magnitude. Chowdhury et al. reported that surface area of durian wood sawdust increased sharply from 1.38 to 221 m^2^/g after carbonization at 550 °C [32]. Elnour et al. also reported that the pyrolysis of date palm wood at 700 °C increased the biochar surface area from 1.0 to 249 m^2^/g [33].

The chemical structure of the carbon in CHF was determined by Raman spectroscopy. The Raman spectrum of CHF (Figure 1b) shows two main bands at 1340 cm^−1^ (disordered carbon, D-band) and 1592 cm^−1^ (graphitic carbon, G-band), which is consistent with the presence of carbonized material [31,34]. The proportion of disordered to graphitic C can be determined quantitatively from the ratio of their band intensities (I_D_/I_G_ or I_1340_/I_1592_). The I_D_/I_G_ for CHF was 1.3, which was within the range of 0.7 (800 °C) to 1.8 (1600 °C) for carbonized lignin [1]. Two minor bands were observed at 2650 cm^−1^ (2D band) and 2889 cm^−1^ (D + G overtone) and are associated with disordered carbon materials with a low degree of graphitization [1].

FTIR spectroscopy was employed to observe chemical changes due to the carbonization of HF to CHF. Figure 1c shows the FTIR spectra of HF, CHF, WF, and graphite. For the WF and HF samples, a strong band at 1034 cm^−1^ was assigned to glycosidic bonds (C–O–C) in the polysaccharides [35]. Bands at 1740 cm^−1^ (C=O stretching of esters and acids), 1375 cm^−1^ (C–H bending), and 898 cm^−1^ (C–H deformation) were also assigned to polysaccharides [35]. Lignin-associated bands were observed at 1235 cm^−1^ (C=O, C–O, and C–C bending in the guaiacyl units), 1325 cm^−1^ (syringyl units with ring breathing), 1460 cm^−1^ (C–H bending), 1515 cm^−1^ (C=C stretching of the aromatic skeleton), and 1630 cm^−1^ (C=C stretching of the aromatic skeleton) [36]. The lignin syringyl/guaiacyl (S/G) ratio of HF was also determined by FTIR spectroscopy to be 1.6, which was higher than for hardwoods, at 1.1–1.2 [37]. The spectrum for CHF was completely different from those of HF and WF, showing: (i) a broad band at 1300–1600 cm^−1^, which was assigned to C=C ring stretching, (ii) a broad band centered at 970 cm^−1^, which was tentatively assigned to C–H in-plane bending and/or the C–O stretching of an aromatic ether, and (iii) a band at 768 cm^−1^, which was assigned to the C–H bending of substituted aromatic rings [38]. The spectrum of the CHF was comparable to that of a commercial graphite sample (Figure 1c).

**Figure 2:**
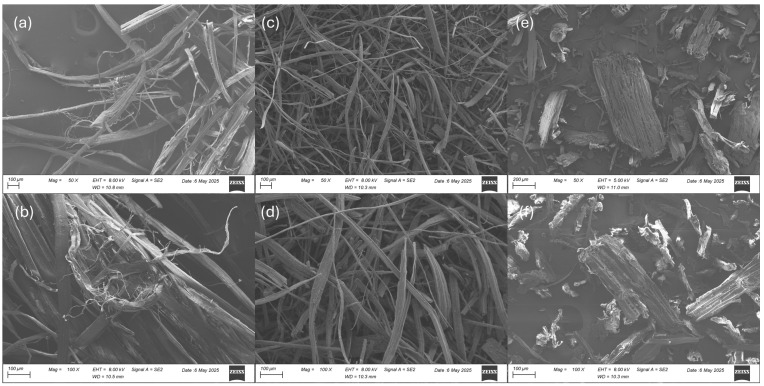
Scanning electron micrographs of (**a**) HF at 50×, (**b**) HF at 100×, (**c**) CHF at 50×, (**d**) CHF at 100×, (**e**) WF at 50×, and (**f**) WF at 100× magnifications.

SEM was performed on the HF, CHF, and WF samples to qualitatively examine their morphology (Figure 2). The HF sample shows long defibrillated and ruptured fibers and fiber bundles (Figure 2a,b). Individual fibers were 14–24 µm in width, while the fiber bundles were 36–107 µm in width and the literature widths were 67 ± 26 µm [9]. The CHF micrographs showed relatively clean, long fibers ranging in width from 7 to 57 µm (Figure 2c,d). The WF micrographs showed cut fibers and cut-fiber bundles with an approximate aspect ratio (length/width) of 2; this is consistent with 40-mesh screened wood particles [18].

### 3.2. Composite Characterization

**Figure 3:**
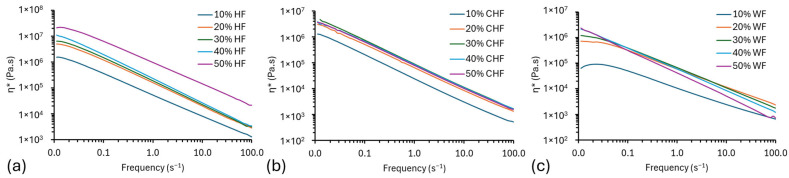
Flow curves (η* versus the frequency) of: (**a**) HF–PRF, (**b**) CHF–PRF, and (**c**) WF–PRF composite blends.

Blends of 10–50% HF, CHF, and WF with PRF were prepared, and the mixtures were analyzed by rheometry [18]. The flow curves (complex viscosity (η*) vs. frequency) of the fiber (HF, CHF, and WF)–PRF resin blends were determined from frequency sweep experiments at 25 °C (Figure 2). The temperature of 25 °C was considered an appropriate ambient operating temperature. Orji et al. [18] observed similar linear shear thinning (pseudoplastic) behavior at PRF > 0.1 Hz but reported that it deviated at low frequencies (<0.1 Hz), and that PRF followed the Carreau–Yasuda model [39]. All the fiber–PRF blends showed pseudoplasticity (shear thinning), as shown in Figure 3. The addition of 10% CHF, HF, and WF to PRF increased the η* value by nearly an order of magnitude at 1 Hz. For HF–PRF blends, the η* value increased with the fiber content (up to 50%), while for the WF and CHF resin blends, η* increased up to a 30% fiber content, then decreased at higher fiber loadings (Table 1). HF showed the greatest increase in η*, followed by CHF and WF. Pseudoplastic behavior influences the ability of the fiber–resin blends to be extruded through a die and their ability to relax adequately after shear has been applied [40]. Furthermore, fiber–resin agglomerates will likely dissociate during extrusion due to the shear forces applied.

The rheological data (η*) were analyzed by fitting to a modified power–law model [18]:

(1) $$\left|\eta^{⋆}\;(\omega )\right|=K(\omega {)}^{n-1}$$

where *n* is the non-Newtonian or flow behavior index, and *K* is the consistency coefficient. Table 1 shows a summary of the results (η* at 1 Hz, *K*, *n* and R^2^) for the power–law models for the fiber–PRF blends. The fiber–PRF formulations yielded goodness-of-fit values (R^2^ > 0.92), except for 10% WF (R^2^ = 0.85), for the fitted power–law models. The WF–PRF and HF–PRF series showed a decrease in *n* values with WF content (10–50%) and HF content (10–40%). This decrease in *n* along with wood content has also been observed with wood–plastic composite melts [41]. However, the CHF–PRF series did not show any trend between the low *n* values (0.104–0.129) and the CHF content.

**Figure 4:**
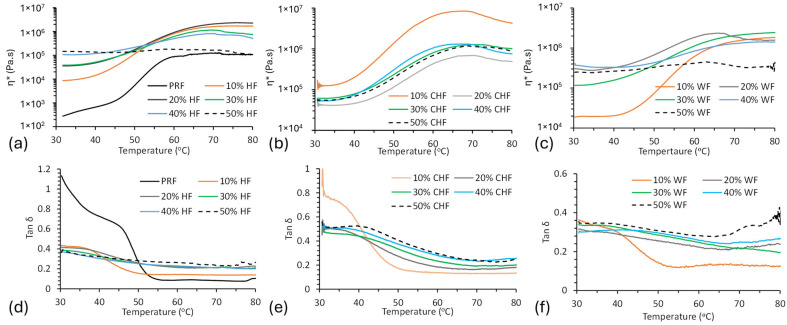
Rheological plots of η* vs. temperature for (**a**) HF–PRF blends, (**b**) CHF–PRF blends, and (**c**) WF–PRF blends, and tan δ vs. temperature for (**d**) HF–PRF blends, (**e**) CHF–PRF blends, and (**f**) WF–PRF blends.

The curing characteristics of PRF resin and fiber–resin blends were monitored for η* as a function of temperature (Figure 4). The PRF resin started to gel at around 44 °C (onset), confirming it to be a cold-setting resin. The authors of [18] observed similar gelation temperatures (37–42 °C) for PRF resin, and vitrification was observed above 60 °C (η* plateaus). Addition of WF, HF, and CHF to the PRF resin led to a progressive increase in gelation onset temperature with fiber content (10–50%).

Plots of tan δ versus temperature for the PRF resin and fiber–resin composite blends are shown in Figure 4. For PRF, tan δ showed a transition, representing gelation, between 45 and 55 °C. These data support the results observed in the η* versus temperature plots (Figure 4a–c).

### 3.3. Thermal Analysis

**Figure 5:**
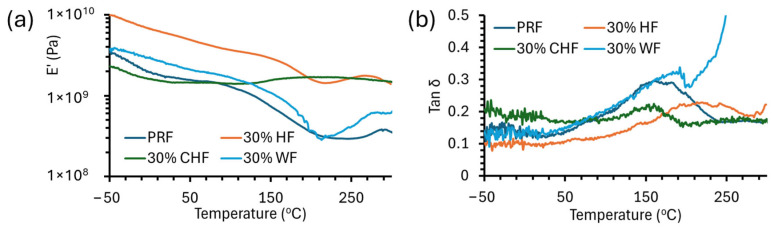
DMA (**a**) storage modulus (E’) and (**b**) tan δ thermograms of PRF and HF–PRF, CHF–PRF, and WF–PRF composites at 30% fiber content.

The viscoelastic properties (tan δ) of cured fiber–PRF composites were determined by DMA in a three-point bending mode, and those of cured PRF resin were determined in compression mode. Figure 5 shows DMA storage modulus (E′) and tan δ thermograms of cured PRF resin and HF–PRF, CHF–PRF and WF–PRF composites at 30% fiber loading. The glass transition (T_g_) of the fiber–PRF composites were determined from (i) the tan δ maxima and (ii) the E′ inflection point, and the results are given in Table 2. The T_g_ of PRF was 168 °C (tan δ max) and is comparable to the literature at 172 °C [18]. The T_g_ demonstrated that the E′ inflection was considerably lower than with the tan δ max method and was not discussed further. With the addition of HF, CHF, and WF fibers, some of the thermograms showed two tan δ maxima peaks. E′ was shown to decrease progressively for the PRF, WF–PRF, and HF–PRF composites with temperatures below the T_g_. The PRF resin and HF–PRF and WF–PRF composites increased in E’ above 200 °C, and this coincided with thermal degradation (see the TGA results). The CHF–PRF composites showed more thermal stability and their E′ did not decrease as drastically as the other fiber types, and, in some instances, increased (e.g., 30% CHF; see Figure 5a). This increase in E′ could be attributable to cross-linking between CHF and PRF resin making the system more rigid. A similar phenomenon was observed, using DMA, in 10% carbonized hemp hurd–polybenzoxazine composites [42]. The E′ generally increased with increasing fiber content, which signifies the reinforcing of the resin structure in the material [43]. The addition of both HF and CHF also increases T_g_ thereby improving the material properties. The authors of [44] have mentioned that there is a need for biobased materials with high T_g_, such as those in this study.

### 3.4. Flexural Properties of Fiber PRF Composites

The flexural modulus (FM) and strength (FS) values of the various compression-molded HF–, WF–, and CHF–PRF composites are given in Table 3. The HF–PRF composites increased in FS from 37 to 63 MPa when changing from 10 to 40% HF content, which clearly shows fiber reinforcement [45,46]. However, the FS at 50% was only 14 MPa, and this may be due to there being insufficient resin content to fully coat and wet the fiber, resulting in a reduction in strength from poor stress transfer. The CHF–PRF composite FS values were between 11 and 18 MPa. The reduction in strength seen from HF to CHF could be attributable to embrittlement of the fibers, due to carbonization, and a decrease in fiber length. The WF–PRF composites showed a similar increase in FS (13 to 63 MPa) when changing from 10 to 40% WF content, as compared to HF–PRF, then decreased at 50% WF content.

**Figure 6:**
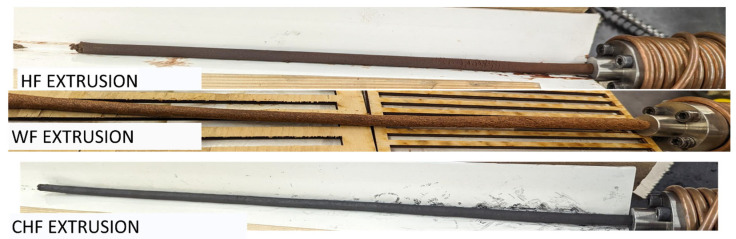
Photographs of the extruded composites of the HF–PRF, WF–PRF, and CHF–PRF composites.

**Figure 7:**
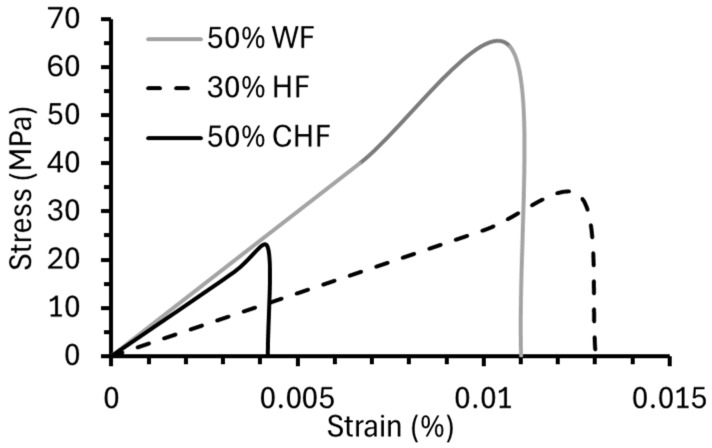
Flexural stress–strain curves for extruded 30% HF–PRF, 50% WF–PRF, and 50% CHF–PRF composites.

The compression molding of WF, HF, and CHF with PRF showed that the composites had good mechanical properties. For their application in AM, the extrudability of fiber–PRF formulations is required. Initial formulations with 50% fiber and 50% PRF were evaluated. The WF–PRF and CHF–PRF blends were successfully extruded, while the HF–PRF mixture would not extrude. Subsequent trials using 30 and 40% HF with PRF were performed, and only the 30% HF formulation was successful. Pictures of the extruded composites are shown in Figure 6. Flexural stress–strain curves for the successfully extruded composite formulations are shown in Figure 7. The FS for the extruded CHF–PRF and WF–PRF composites were 22.7 ± 1.6 and 65.4 ± 2.7 MPa, respectively. The FS values of the CHF and WF composite samples were higher than their corresponding compression-molded samples. The increase in FS is likely due to the better packing and fiber alignment caused by the extrusion process. Similarly, Frone et al. reported that extruded and 3D printed cellulose nanocomposites have better mechanical properties than compression-molded samples [47]. In contrast, the HF–PRF extruded composite had a lower FS, at 33.0 ± 1.8 MPa, than the compression-molded sample at 59 MPa, which could be attributable to the clumping of long HF fibers, reducing its strength. The FM of the extruded 30% HF–PRF, 50% CHF–PRF, and 50% WF–PRF composites were 2.6 ± 0.3, 5.4 ± 0.4, and 6.0 ± 0.2 GPa, respectively. In comparison, the compression-molded 50% CHF–PRF composite was only 1.5 GPa. These results show that the CHF–PRF composite had good mechanical properties after extrusion because of improved packing from the increased shear forces and fiber alignment and could offer suitable reinforcement for AM applications [48,49,50]. The compression-molded and extruded 50% WF–PRF composites had comparable FM values.

### 3.5. Dimensional Stability of the Composites

The water-soak stability (water absorption (WA) and thickness swell (TS)) of the compression-molded fiber–PRF composites were assessed at 24 and 72 h (Table 4). The TS values of 50% WF, HF, and CHF after 72 h were 26, 13, and 5.9%, respectively. The WA for 50% WF, HF, and CHF after 72 h were 30, 30, and 25%, respectively. The CHF composites were shown to have better dimensional stability than the WF and HF composites, and this is most likely attributable to CHF being hydrophobic. Dimensional stability is important for the product performance of materials over time [17]. Chen et al. [51] and Lei et al. [43] reported that CF-reinforced composites have good dimensional stability, due to their better resistance to biological, chemical, and thermal degradation.

### 3.6. Thermal Stability

**Figure 8:**
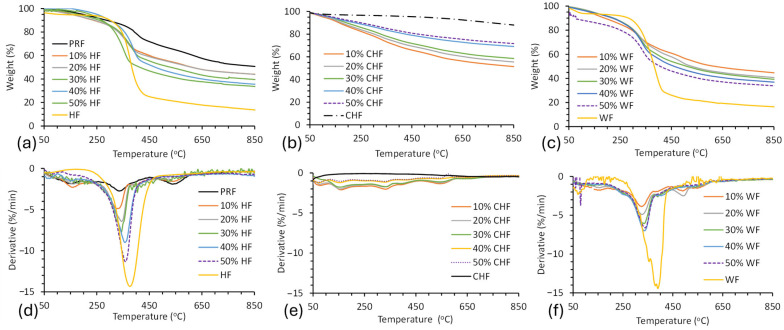
TGA of (**a**) HF and PRF composites, (**b**) CHF and PRF composites, and (**c**) WF and PRF composites and DTG of (**d**) HF and PRF composites, (**e**) CHF and PRF composites, and (**f**) WF and PRF composites.

TGA was performed to determine the thermal stability of the fibers, cured PRF resin, and fiber–PRF composites (Figure 8). The onset temperature (T_onset_), residual weight at 850 °C, and DTG maxima temperature (DTG_max_) for the fibers, cured PRF resin, and fiber–PRF composites are given in Table 5. The thermograms show a small amount of weight loss below 200 °C, which is attributable to (i) moisture, (ii) volatiles, and (iii) degradation products due to cross-linking and auto-condensation reactions. Bouajila et al. observed the release of water, methanol, formaldehyde, and phenolics from cured PRF resin below 200 °C by TGA-mass spectrometry and TGA-FTIR experiments [52]. Water can be released from pores within the resin and can then be produced by condensation reactions due to vitrification. Formaldehyde can be released when dimethylene ether linkages are converted to methylene bridges. The T_onset_ for the cured PRF resin was 208 °C, with a residual weight of 69% at 500 °C and 48% at 850 °C. The T_onset_ for WF–PRF and HF–PRF composites ranged from 215–218 °C and 234–312 °C, respectively. These results are characteristic of lignocellulosic fiber degradation [53]. The T_onset_ for the HF and WF composites was shown to reduce along with HF and WF content. These findings agree with the literature [17,54]. Furthermore, the residual mass at 850 °C for the HF and WF composites decreased with fiber content, supporting their lower thermal performance. The T_onset_ of CHF–PRF composites were difficult to determine due to a gradual change in weight with temperature due to their improved thermal stability. The curves for the CHF composites showed improved thermal performance with CHF content, as judged by the residual mass at 850 °C, and this was also seen for carbonaceous-based composite materials [30,55]. The CHF was shown to have a residual weight of 95% at 500 °C and 88% at 850 °C, while the 50% CHF–PRF composite was 72%. The high thermal stability of CHF was attributable to its high C content of 88% [1,56]. In comparison, the thermal stability of the CF–epoxy composite had a residual weight of 77% at 900 °C [56].

## 4. Conclusions

Long carbonized hemp fiber (CHF) was successfully prepared from hemp fiber (HF) via a slow activation and pyrolysis process to yield a CHF with high carbon content. The CHF contained both amorphous and graphitic carbon structures that were comparable to carbonized lignin. Composites of WF, HF, and CHF at a 10–50% content with PRF were prepared successfully by compression molding, and the mechanical, rheological, and thermal properties were investigated and compared. The wet composite blends showed good rheological behavior that could make them suitable for extrusion-based processes and products. The addition of CHF improved the thermal stability of the composites with increasing content, while HF and WF reduced thermal stability progressively. The CHF–PRF composites were shown to have better dimensional stability in a water-soak test, due to their hydrophobic character, than the HF–PRF and WF–PRF composites. For the compression-molded composites, the addition of CHF (10–50%) did not significantly improve their flexural properties, in contrast to the HF and WF composites, which had a maximum strength of 63 MPa and modulus of 6.6 GPa. The extrusions of 50% CHF, 50% WF, and 30% HF in PRF were successful. The extruded CHF–PRF showed a good flexural modulus of 5.4 GPa, in contrast to the compression-molded sample (1.5 GPa). This modulus improvement is most likely due to better blending and material compaction from extrusion. The extruded WF–PRF had a flexural modulus of 6.0 GPa. These results show that CHF-based composites, as well as HF- and WF-based thermoset composites, can be extruded at high fiber loadings for use in extruded profiled products and may potentially be used in additive manufacturing applications in construction. Future work will focus on evaluating these extrudable formulations on a custom-built 3D printer for printability and interlayer bonding.

## Figures and Tables

**Table 1:** Complex viscosity (η*) and power–law-fitted model parameters (*K* and *n*) for fiber–resin composite blends.

Sample	η* at 1 Hz (kPa.s)	*K* (kPa.s)	*n*	R^2^
PRF	3.76			
10% CHF	24.3	25.4	0.105	0.997
20% CHF	65.2	66.4	0.127	0.998
30% CHF	90.8	93.2	0.104	0.999
40% CHF	85.7	85.2	0.129	0.993
50% CHF	80.6	81.6	0.119	0.996
10% HF	54.1	54.4	0.192	0.986
20% HF	162	153	0.136	0.975
30% HF	191	180	0.105	0.975
40% HF	233	224	0.081	0.991
50% HF	979	932	0.196	0.963
10% WF	10.6	10.3	0.403	0.846
20% WF	58.3	56.6	0.322	0.927
30% WF	66.3	61.5	0.247	0.962
40% WF	58.9	56.7	0.173	0.999
50% WF	41.6	40.8	0.100	0.999

**Table 2:** Storage modulus (E′) and T_g_ values of the composites, as determined by DMA.

Sample	T_g_ by tan δ (°C)	T_g_ by E′ Inflection (°C)	E′ (GPa) at −40 °C	E′ (GPa) at 20 °C
PRF	168	135	3.16	1.66
10% HF	200	163	5.70	3.53
20% HF	197	160	6.93	4.21
30% HF	220	174	9.26	5.97
40% HF	190	173	8.21	5.31
50% HF	216	190	2.51	5.32
10% CHF	172, 225	165	4.60	2.38
20% CHF	190	165	3.86	2.13
30% CHF	161	n.d.	2.20	1.49
40% CHF	241	n.d	2.70	2.12
50% CHF	220	n.d.	2.57	1.65
10% WF	228	134	2.04	0.895
20% WF	182, 213	130	7.54	3.54
30% WF	186	135	3.67	2.46
40% WF	181, 278	130	6.26	3.88
50% WF	280	130, 190	4.65	2.25

**Table 3:** Flexural strength and modulus of the compression-molded fiber–PRF composites.

Sample	Flexural Strength (MPa)	Flexural Modulus (GPa)
10% HF	36.8 ^c^ (1.8) *	2.43 ^d^ (0.14) *
20% HF	47.7 ^b^ (1.6)	2.36 ^c^ (0.17)
30% HF	59.2 ^a^ (1.9)	3.64 ^c^ (0.13)
40% HF	63.1 ^a^ (3.7)	4.55 ^b^ (0.21)
50% HF	13.8 ^de^ (0.7)	0.536 ^fg^ (0.07)
10% CHF	11.4 ^f^ (0.8)	0.514 ^h^ (0.04)
20% CHF	16.7 ^de^ (1.0)	0.613 ^gh^ (0.06)
30% CHF	18.5 ^d^ (1.0)	0.939 ^fgh^ (0.07)
40% CHF	11.3 ^f^ (0.4)	0.967 ^fgh^ (0.048)
50% CHF	15.8 ^de^ (0.7)	1.50 ^ef^ (0.11)
10% WF	12.9 ^de^ (1.4)	0.956 ^fgh^ (0.100)
20% WF	40.3 ^c^ (1.6)	3.43 ^c^ (0.25)
30% WF	51.4 ^b^ (2.2)	4.95 ^b^ (0.19)
40% WF	63.2 ^a^ (2.4)	6.09 ^a^ (0.20)
50% WF	43.8 ^b^ (3.5)	6.62 ^a^ (0.58)

* Values are mean values, with standard deviation in parenthesis. Where necessary, the standard deviation has been rounded up to the mean’s reported precision. Statistical differences in the results were measured via ANOVA test (*p*-value < 0.05) and are shown by superscript letters (a–h).

**Table 4:** Water-soak tests (WA and TS) of the compression-molded composites.

Sample	24 h WA (%)	72 h WA (%)	24 h TS (%)	72 h TS (%)
10% HF	22 (0.2)	22 (1.6)	14 (0.5)	22 (0.8)
20% HF	20 (1.8)	21 (0.7)	21 (2.6)	22 (3.4)
30% HF	18 (0.9)	19 (4.8)	13 (7.1)	18 (8.7)
40% HF	20 (0.4)	22 (1.9)	14 (1.8)	15 (1.0)
50% HF	26 (3.6)	30 (0.8)	10.7 (2.8)	13 (1.6)
10% CHF	17 (1.4)	17 (2.1)	7.2 (5.8)	11.3 (2.1)
20% CHF	18 (5.2)	21 (2.4)	2.6 (1.1)	7.2 (0.8)
30% CHF	19 (3.4)	21 (2.1)	1.4 (0.9)	2.5 (1.2)
40% CHF	18 (1.1)	21 (2.6)	0.9 (0.7)	3 (1.4)
50% CHF	23 (3.3)	25 (1.5)	4.1 (3.2)	5.9 (0.1)
10% WF	19 (1.3)	22 (1.1)	12 (3.0)	14 (0.9)
20% WF	22 (1.4)	23 (1.1)	14 (1.3)	19 (2.0)
30% WF	18 (0.64)	21 (0.52)	18 (2.1)	19 (0.9)
40% WF	20 (1.8)	23 (1.5)	22 (4.1)	24 (1.4)
50% WF	26 (3.9)	30 (3.2)	25 (3.7)	26 (2.3)

Values are mean values, with standard deviation in parenthesis. Where necessary, the standard deviation has been rounded up to the mean’s reported precision.

**Table 5:** Thermal stability results for HF, CHF, WF, and their PRF composites, as determined by TGA.

Sample	DTG_max_ (°C)	T_onset_ (°C)	Residual at 500 °C (%)	Residual at 850 °C (%)
PRF	335	208	69	49
HF	412	371	24	15
10% HF	335	312	55	41
20% HF	342	308	49	40
30% HF	342	296	47	36
40% HF	361	263	42	29
50% HF	362	234	38	25
CHF	893		95	88
10% CHF	334	310	63	51
20% CHF	328	313	67	55
30% CHF	325	360	70	59
40% CHF	328	379	77	69
50% CHF	576	382	79	72
WF	389	259	23	16
10% WF	336	289	57	45
20% WF	331	281	52	41
30% WF	350	247	51	39
40% WF	340	220	47	37
50% WF	345	215	43	34

## Data Availability

The original contributions presented in this study are included in the article. Further inquiries can be directed to the corresponding author.
